# Investigation on the Relationship between Biodiversity and Linguistic Diversity in China and Its Formation Mechanism

**DOI:** 10.3390/ijerph19095538

**Published:** 2022-05-03

**Authors:** Xuliang Zhang, Zhanting Bu, Hongrun Ju, Yibo Jing

**Affiliations:** 1 School of Tourism and Geography Science, Qingdao University, Qingdao 266071, China; jhr621@126.com (H.J.); qddxyibo@163.com (Y.J.); 2 School of Foreign Languages, Qingdao University, Qingdao 266071, China; harrybu@163.com

**Keywords:** Chinese dialects, linguistic diversity, biodiversity, regional correlation

## Abstract

Previous studies have demonstrated that countries, biodiversity hotspots, wildness areas, and islands with high biodiversity also have high linguistic diversity, while the regional correlation between phonetic, lexical, and grammatical diversity within a particular kind of language and biodiversity has not been verified. Based on the methods of GIS visualization and Spearman correlation coefficient, the regional differences and correlations between linguistic diversity and biodiversity in China are investigated in this paper using the numbers of plant species, animal species, Chinese dialects, and the data of phonetic, lexical, and grammatical diversity of Chinese dialects. The results reveal the positive regional correlations between the diversity of Chinese dialects, as well as the phonetic, lexical, and grammatical diversity of Chinese dialects and biodiversity. In addition, the regional correlation between linguistic diversity and plant diversity is stronger than that between linguistic diversity and animal diversity. The diversity of Chinese dialects is being weakened by the industrialization and urbanization. Furthermore, some countermeasures to protect linguistic diversity are proposed, such as protecting biodiversity and small communities, as well as promoting national language resource protection projects.

## 1. Introduction

Language has the functions of information transmission, society construction, and social cognition [1,2,3]. Linguistic diversity indicates the typological diversity of languages and dialects, and the diversity of phonetics, lexicon, and grammar within languages [4,5]. It has become a human consensus to protect linguistic diversity [6,7].

Languages exhibit the trends of differentiation, unification, and extinction [8]. The extinction of languages is induced by such factors as globalization, industrialization, urbanization, the distinction of biodiversity, impacts of mainstream culture to non-mainstream culture, and population migration. There were approximately 20,000 kinds of languages in pre-agricultural society, while today approximately 6912 kinds of languages exist [9]. Furthermore, one-third to one-half of all existing languages are considered endangered [10].

There is a positive regional correlation between linguistic diversity and biodiversity. Generally, the areas with high biodiversity possess high linguistic diversity [11,12]. The acreage of the total 35 biodiversity hotspots only accounts for 2.3% of the global land while having more than 50% of the world’s vascular plant species, at least 43% of the vertebrate species, and 3202 kinds of languages. There are five wildness areas with high biodiversity each of which has an acreage of more than 10,000 km^2^ and their natural habitat loss of is less than 30%. The total acreage of these five wildness areas accounts for 6.1% of the global land, but they have 17% of the world’s total vascular plant species, 8% of the vertebrate species, and 1622 kinds of languages [13]. The area of 238 World Natural Heritage Sites in 96 countries takes up 1% of the global land, but they have 445 kinds of languages [14].

Islands also have high biodiversity and linguistic diversity. There are more than 180,000 islands in the world with a total acreage of 782.1 × 10^4^ km^2^, accounting for 5.3% of the global land. These islands contain 48,331, 1947, and 388 species of flowering plants, birds, and rodents, respectively, and 2551 kinds of languages, accounting for 17%, 19%, 17%, and 27% of all species or kinds around the world, respectively [15].

With the increasing awareness of linguistic diversity conservation, linguists have analyzed the regional correlation between biodiversity and linguistic diversity using the principle of the relationship between creatures and environments [16,17,18,19]. However, most previous studies have only revealed the regional correlation between linguistic diversity and biodiversity on the level of languages and in large-scale regions while neglecting the correlation on the level of phonetic, lexical, and grammatical diversity of languages. Meanwhile, the formation mechanism of the regional correlation has not been thoroughly investigated [20,21].

Biodiversity is formed by the variation and evolution of creatures to adapt to the environment [22]. The regional difference in biodiversity has been established through the diversity and regional differences in the environment. Generally, biodiversity is high in hot and humid areas. Two-thirds of the total primate species in the world are in Brazil, Zaire, Madagascar, and Indonesia; more than half of tropical rainforest with high biodiversity is distributed in Brazil, Zaire, and Indonesia [23]. The places with large topography differences have more diverse environments and higher biodiversity when latitudinal and sea-land locations are roughly the same.

There are abundant animal and plant species and kinds of vegetation in China, including 45,788 species of plants that belong to 9 phylums of Anthocerotophyta, Bryophyta, Chlorophyta, Rhodophyta, Lycopodiophyta, Marchantiophyta, Pteridophyta, Gymnospermae, and Angiospermae, as well as 60,081 species of animals that belong to 17 phylums of Annelida, Arthropoda, Brachiopoda, Bryozoa, Chordata, Cnidaria, Echinodermata, Echiura, Entoprocta, Adelochorda, Mollusca, Myxozoa, Nematoda, Nemertea, Phoronida, Porifera, and Sipuncula [24]. The high biodiversity in China and its regional difference are formed by vast territory, latitude and longitude spans, and huge terrain fluctuations [25].

Chinese dialects are of various types and are spoken by 1206.89 million people. The formation of various Chinese dialects with phonetic, lexical, and grammatical differences has been influenced by natural and human factors of many speakers, wide distribution, inconvenient transportation, regime separation, terrain barrier, low productivity in the early society, and little language communication among people in different regions. Chinese dialects are divided into regional dialects and social dialects. Regional dialects refer to the varieties and branches of Chinese in different areas formed due to regional isolation and regional imbalance of Chinese development. Social dialects reflect the varieties of Chinese in the same region influenced by differences of residents in the profession, age, gender, and education [26].

Regional differences in the phonetic, lexical, and grammatical diversity of Chinese dialects have been broadly researched [27], but there are few studies on the regional correlation between the diversity of Chinese dialects and biodiversity.

The world is encountering a crisis of language loss that rivals, or exceeds, the rate of biodiversity loss [10]. The situation of language endangerment is also severe in China. Specifically, approximately 50 percent of China’s minority languages are endangered to varying degrees, and some minority languages with fewer speakers are gradually dying off and threatened by dominant languages [28]. Exploring whether there is a positive regional correlation between linguistic diversity of Chinese dialects and biodiversity in China and its formation mechanism can contribute to the protection of biodiversity and linguistic diversity in China.

## 2. Data and Methods

The data collected in this study contain the numbers of Chinese dialects; the phonetic, lexical, and grammatical diversity of Chinese dialects; and the numbers of plant species and animal species in 33 provincial administrative regions (PARs) of China, except for Tibetan Autonomous Region. The numbers of Chinese dialects were derived from the reference [29]. The total numbers of variants of the 160 phonologies, the phonetics of the 32 special characters, and the source and change (region difference) of 13 important phonetic values of Chinese dialects, as well as the numbers of the regional combinations of the division of the *Rù* tone, which reflect the characters of the phonological diversity of Chinese dialects of the 33 PARs, were obtained from 205 maps in *Linguistic Atlas of Chinese Dialects · Phonetics* [30]. The total numbers of the dialectical expressions for the 203 concepts (Chinese words) and the numbers of the dialectical expressions for the concept (Chinese word) “father”, which demonstrate the characters of the lexical diversity of Chinese dialects of the 33 PARs, were acquired from 203 maps in *Linguistic Atlas of Chinese Dialects · Lexicon* [31]. The total numbers of the dialectical expressions for the 102 Chinese grammatical words, morphology, and syntax, and the numbers of the regional combinations of the eight surveyed kinship terms using reduplication, such as grandpa, grandma, dad, mom, elder brother, elder sister, younger brother, and younger sister, which reveal the characters of the grammatical diversity of Chinese dialects, were derived from 102 maps of *Linguistic Atlas of Chinese Dialects · Grammar* [32]. The numbers of plant species and animal species were collected from the website of Species 2000 China Node “Catalogue of Life China: 2021 Annual Checklist” [24].

The data were input into the software ArcGIS10.2 developed by Environmental Systems Research Institute, USA, and divided into five grades using the natural discontinuity point method. The distribution figures of the kind numbers of Chinese dialects, the phonetic, lexical, and grammatical diversity of Chinese dialects, the numbers of the regional combinations of the division of the *Rù* tone, the numbers of Chinese dialects presentations of the word “father”, the numbers of the regional combinations of the eight surveyed kinship terms using reduplication, the total numbers of animal and plant species, the numbers of plant species, and the numbers of animal species were drawn by the method of the hypsometric layer. The regional differences in Chinese dialect diversity, and biodiversity in China were analyzed.

Spearman correlation coefficient is a non-parametric index to measure the dependence of two variables. It uses the monotone equation to evaluate the correlation of the two statistical variables, while the data are not required to conform to normal distribution and linear relation [33,34]. Spearman correlation coefficient (*ρ*) was adopted to validate the regional positive correlation between biodiversity and the diversity of Chinese dialect and their phonetic, lexical, and grammatical diversity:

(1) $$\rho =\frac{\sum_{i=0}^{n}\left(x_{i}-\bar{x}\right)\left(y_{i}-\bar{y}\right)}{\sqrt{\sum_{i=0}^{n}{\left(x_{i}-\bar{x}\right)}^{2}\sum_{i=0}^{n}{\left(y_{i}-\bar{y}\right)}^{2}}}$$

where *ρ* denotes the correlation coefficient; *n* represents the capacity of samples; *x_i_* indicates the rising rank of species numbers of animals and plants, plants, or animals; $\bar{x}$ designates the arithmetic mean value of *x_i_*; *y_i_* is the rank of the kind numbers of Chinese dialects, the rank of total variant numbers of the 205 phonologies, the pronunciations of special characters and important phonetic values of Chinese dialects, the rank of the total numbers of the dialectal expressions for 203 Chinese words, the rank of the total numbers of the dialectal expressions for 102 grammatical words, morphology and syntax, the rank of the numbers of the regional combinations of the division of the *Rù* tone, the rank of the numbers of the Chinese dialectal expressions for word “father”, and the rank of the numbers of the regional combinations of the 8 surveyed kinship terms using reduplication in the 33 PARs; and $\bar{y}$ stands for the arithmetic mean value of *y_i_* (Table 1). Moreover, the formation of the regional correlation between linguistic diversity and biodiversity was also discussed, and measures were proposed to protect the diversity of Chinese dialects.

## 3. Results

### 3.1. Diversity and Regional Differences of Chinese Dialects

Following the regional differences in Chinese dialects’ phonetics, lexicon, and grammar, the distributing areas of Chinese dialects are divided into 10 districts and 97 dialect slices, and the Mandarin district is divided into 8 Mandarin sub-dialect districts (Table 2) [26].

**Figure 1:**
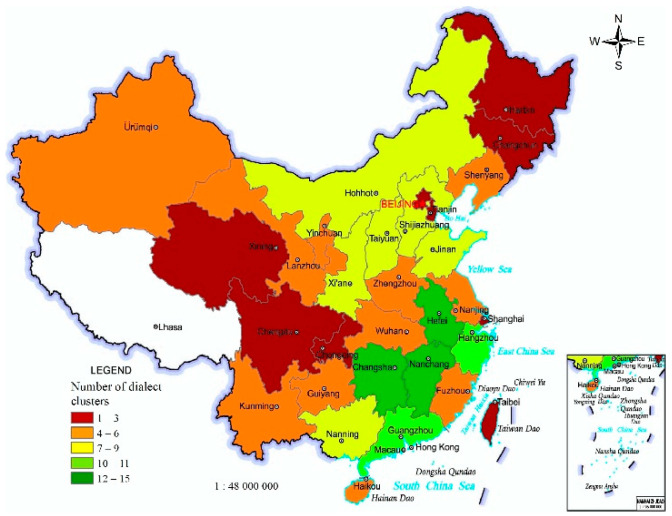
Number of Chinese dialects in the 33 PARs of China.

Chinese dialects in different slices exhibit regional differences in phonetics, lexicon, and grammar. A dialect can represent a kind of Chinese dialect [29]. The number of Chinese dialect slices in the 33 PARs is roughly characterized by regional differences of “many in the south-east, fewer in the north-west, and fewest in the south-west and north-east” (Figure 1).

In this study, 77 initials, 84 finals, 38 tones, and 4 combinations of initials and finals were investigated (Appendix A Table A1). Meanwhile, 160 maps of correspondences and exemplar characters, 32 maps of special characters, and 13 maps of phonetic values were drawn to reveal the regional differences and historical evolutions of phonologies, pronunciations of special characters, and phonetic values of Chinese dialects. Maps of correspondences demonstrate the regional differences and historical evolutions of phonologies, namely, the types and conditioning factors of preservation, split, merge, variation, and regulation of particular phonological categories, such as the division of the *Píng* tone, the evolutions of *Rù* tone and *Qièyùn* voiced obstruent initials, and the distinction between the *Qièyùn* dental nasal and lateral initials. Maps of exemplar characters imply the regional differences in regular pronunciations of exemplar characters. Maps of special characters reflect the regional differences of the sources and evolutions of special characters’ pronunciations, such as the tone of *bí* (nose), the initial in *tŏng* (bucket), and the final in *dă* (beat). Maps of phonetic values suggest the regional differences of some representative or important phonetic values of Chinese dialects [35].

**Figure 2:**
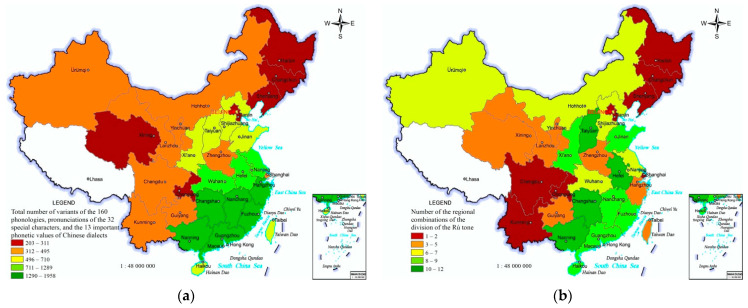
Distribution of phonetic diversity of Chinese dialects in China. (**a**) Total number of sources and variants of the 160 phonologies, pronunciations of the 32 special characters, and the 13 important phonetic values of Chinese dialects. (**b**) Number of the regional combinations of the division of the *Rù* tone of Chinese dialects.

The total number of sources and variations of 160 phonologies, pronunciations of 32 special characters, and 13 important phonetic values in the 33 PARs can indicate the regional differences in phonetic diversity of Chinese dialects. It presents roughly regional differences of “many in the south-east, fewer in the north-west and south-west, and fewest in the north-east” (Figure 2a). The *Rù* tone has 6 sorts of divisions, 1, 2, 3, 4, 5, or 6 divisions, and 35 types of regional combinations [30]. The number of the regional combinations of the division of the *Rù* tone is a representative indicator reflecting the regional differences in phonetic diversity. The regional difference has the characters of “many in the south-east, fewer in the north-west, and fewest in the south-west and north-east” (Figure 2b).

The words of Chinese dialects are divided into four types of homonyms words, synonyms words, special words, and a type of idioms, proverbs, and two-part allegorical sayings of Chinese dialects. They are different in the origin, meaning, word-formation, value, and phonetic change. Particularly, the differences in origin result in the homologous words and heterologous words of Chinese dialects. The various words of Chinese dialects are different in their meaning breadth, extended meanings, grammar, and rhetoric. The words of Chinese dialects have been formed in various ways of overlapping, affixation, single, or polyphony. They are also different in morpheme order, alliteration, assonance, derivative sound, and embedded sound [36].

The regional differences and historical evolutions of the dialectical expressions for the 203 surveyed Chinese words are illustrated in 188 semantics maps, 6 morphemes maps, 4 distinctions maps, and 5 summary maps [31]. The semantics maps reveal the specific statement of words in different Chinese dialects, such as *taìyáng* (the Sun), *jīntiān* (today), *chī* (eat), and *rè* (hot). The morphemes maps demonstrate the meanings of various morphemes in different Chinese dialects, such as the meanings of *shŏu* (hand), *jiăo* (foot), and *fáng* (house). The distinctions maps suggest whether the same word forms are used to express different concepts in different Chinese dialects, such as the similarities and differences between *wàisūn* (daughter’s son) and *wàisheng* (sister’s son), and *hē* (drink) and *chī* (eat). The summary maps indicate the results of further synthesizing and analyzing the existing materials, such as the idioms referring to “the moon” as a person, the terms for “penis” derived from animal names, and the euphemisms for pig’s tongue. The 203 Chinese words are composed of 95 nouns, 29 adjectives, 53 verbs, 3 numerals, 2 adverbs, 10 quantifiers, and 11 special words or morphemes (Appendix A Table A2).

**Figure 3:**
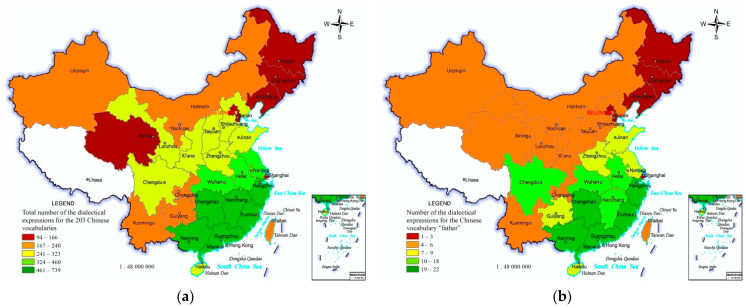
Distribution of lexical diversity of Chinese dialects in China. (**a**) Total number of the dialectical expressions for the 203 Chinese words. (**b**) Number of Chinese dialects expressions of the Chinese word “father”.

The total number of the dialectical expressions for the 203 Chinese words in the 33 PARs can reflect the regional differences in the lexical diversity of Chinese dialects. The total number exhibits the roughly regional differential characteristics of “most in the south-east, more in the central section, fewer in north-west and south-west, and fewest in north-east” (Figure 3a).

A Chinese dialectal word with the same morpheme is one kind of dialectal expression regardless of the same phonetics. However, it will be another dialectal expression if the morpheme changes and the phonetics remain the same. A Chinese word with the same phonetics but different morphemes is considered a different dialectical expression in different regions. Most of the 203 surveyed words have multiple dialectical expressions in different regions. The 10 nouns—*father*, *mother*, *sunflower*, *wife*, *child*, *village*, *boar* (used for breeding), *beautiful*, *paternal grandmother*, and *daughter* (direct address)—have the most dialectical expressions, among which, 5 nouns are frequently and widely used kinship addresses. The 10 words with the least dialectical expressions contain 2 nouns, *rén* (person) and *huŏkàng* (heated brick bed); 2 numerals, *one* and *two*; 2 adjectives, *duō* (many) and *hòu* (thick); and 4 special words or morphemes, a semantic range of euphemisms for *pig’s liver*, *wénzi* (mosquito), *cháng* (long), *shŏu* (hand), and *jiăo* (foot). “*Heated brick bed*” is applied only by Chinese northerners. The dialectical expressions of quantifiers and adjectives, though used frequently, are few because the meanings of quantifiers are fixed and the main functions of adjectives are modification. Moreover, the dialectical expressions of special words or morphemes are also few since they are not frequently used.

“*Father*” is the Chinese word with the most dialectical expressions and is a representative indicator reflecting the regional differences in lexical diversity of Chinese dialects. The number of dialectal expressions for “*father*” in the 33 PARs has the roughly regional differential characteristics of “many in the south-east, fewer in the north-west, and fewest in the north-east” (Figure 3b).

The regional differences and historical evolutions of dialectical expressions of the 102 surveyed Chinese grammatical words, morphology, and syntax (Appendix A Table A3) are demonstrated in 51 structure maps (29 maps of standard Chinese forms and 22 maps of dialect forms), 39 grammatical word maps, and 12 summary maps. The structure maps indicate the forms of grammar, morphology, and syntax structure at the lexical, phrasal, and sentential levels in different Chinese dialects. The maps of standard Chinese forms present the regional differences of grammatical structures in the mandarin Chinese at both the lexical level, and the phrasal or sentential level, such as the suffix in *zhuōzi* (table), the perfective aspect with a substantive object (*wŏ chī le yī wăn fàn*, I ate a bowl of rice), the disposal construction (*tā bă wăn dă pòle*, he broke the bowl), and the comparative (*wŏ bĭ tā dà*, I am older than him). The maps of dialect forms reveal the regional differences of grammatical phenomena only in Chinese dialects, such as quantifiers as demonstratives (*zhī jī sĭle*, the hen/cock is dead), the noun suffix *jiăn* (son or daughter), and *xiān* as postposition marking preceding action in *nĭ qù xiān* (you go first). The grammatical word maps suggest the diversity and regional differences of dialectical expressions of pronouns, adverbs, and expletives with stronger grammatical attributes, such as the first-person singular pronoun *wŏ* (I), degree adverb *hĕn* (very) for adjectives, the possessive particle *de* (*wŏde dōng xī*, my things), perfective aspect *le* without object (*tā laíle*, he is coming), and the passive marker *bèi* (*yīfu bèi tōu zŏule*, clothes are stolen). The summary maps reflect the diversity and regional differences of some comprehensive grammatical phenomena of word-formations and grammatical functions in Chinese dialects, such as the plural forms of personal pronouns, negative word types, diminunatives, and animal gender constructions [32].

**Figure 4:**
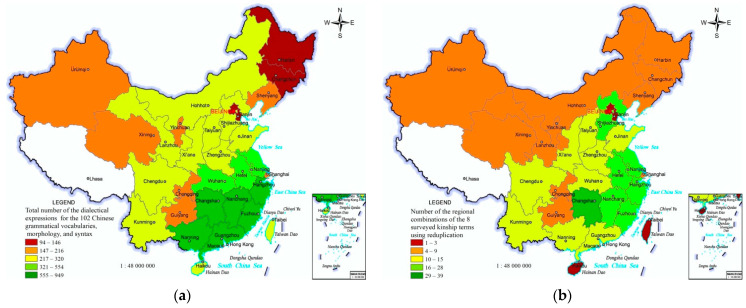
Distribution of grammatical diversity of Chinese dialects. (**a**) Total number of the dialectical expressions for the 102 Chinese grammatical words, morphology, and syntax. (**b**) Number of the regional combinations of the 8 surveyed kinship terms using reduplication.

The regional differences in grammatical diversity can be demonstrated by the total number of the dialectical expressions for the 102 surveyed Chinese grammatical words, morphology, and syntax in the 33 PARs. The total number has the regional differential characteristics of “many in the south-east, fewer in the north-west, and fewest in the north-east in China”. The grammatical diversity of Chinese dialects in Yunnan, Guizhou, Sichuan, and Chongqing in the southwestern Mandarin district is lower than that in the south-eastern PARs, while there are many kinds of ethnic minority languages in these three provinces (Figure 4a).

In Chinese dialects, *paternal grandfather*, *paternal grandmother*, *father*, *mother*, *elder brother*, *elder sister*, *younger brother*, and *younger sister* are frequently-used kinship terms. There are 83 regional combinations of the 8 kinship terms using reduplication, including 8, 7, 6, 5, 4, 3, 2, or 1 kinship terms using reduplication. The number of the regional combinations in the 33 PARs is one of the representative indicators reflecting the regional differences of grammatical diversity of Chinese dialects, which have the regional differential characteristics of “many in south-eastern and fewer in north-western and north-eastern” (Figure 4b).

### 3.2. China’s Regional Differences in Biodiversity

**Figure 5:**
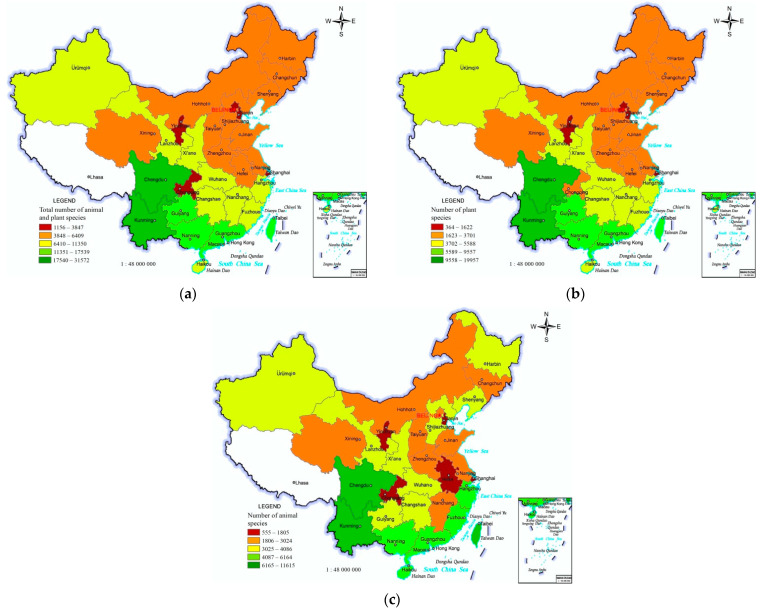
The numbers of species of animals and plants, plants, and animals in the PARs of China. (**a**) Total number of animal and plant species. (**b**) Number of plant species. (**c**) Number of animal species.

The species of plants and the species of animals as well as their sum are the main indicators demonstrating the regional biodiversity at a creature species level. The plant and animal species have the general regional differential characteristics of “many in south-western and fewer in north-eastern”. Many southern PARs have excessive plant and animal species, such as 31,572 in Yunnan and 22,724 in Sichuan. The plant and animal species in Guizhou, Guangxi, Guangdong, Fujian, Zhejiang, Hainan, and Taiwan also exceed 10,000. There are fewer plant and animal species in the northern and southern PARs with small areas, high population density, and urbanization (Figure 5a).

The plant species have a similar regional difference as the total plant and animal species, decreasing from south-western to north-eastern. There are more plant species in the southern PARs (such as 19,957 in Yunnan and 13,494 in Sichuan) and fewer plant species in the southern PARs with small areas and high urbanization (such as Jiangsu, Anhui, Chongqing, Shanghai, Hong Kong, and Macao) (Figure 5b).

The animal species also have a similar regional difference of decreasing from the south-east to the north-east. The southern PARs have many animal species. For example, Yunnan, Taiwan, and Sichuan possess 16,157, 10,547, and 9230 species, respectively. Most northern PARs have fewer than 4000 animal species, except for Shannxi and Xinjiang. Furthermore, the animal species are still fewer in the southern PARs with small areas, high population density, and urbanization, such as Jiangsu, Anhui, Chongqing, Shanghai, Hong Kong, and Macao (Figure 5c).

China’s regional differences in biodiversity are mainly influenced by latitude, distance to the sea, and terrain fluctuation. The southern PARs of tropical and subtropical monsoon climate zone possess many plant and animal species owing to their low latitude, hot and humid climate, and large terrain fluctuation. The plant and animal species decrease from the eastern monsoon climate zones to the western non-monsoon climate zones in the temperate zone due to the decline of precipitation. The biodiversity in mountainous areas and plateaus with large terrain fluctuation is high. The plant and animal species are most numerous in Yunnan, Sichuan, and Guizhou of the subtropical Hengduan Mountainous Area, with numerous peaks and valleys. The elevation of many peaks is around 4000 m, some even higher than 5000 m, and the relative height of peaks and valleys exceeds 2000 m. The climates in the valleys exhibit the hot, humid characteristics influenced by the southwest monsoon, and dramatic vertical variations. The Hengduan Mountainous Area is one of the most imperative forming and differentiating centers of plant and animal species. The biodiversity of northwestern interior grasslands and deserts of China is low due to all-year-round drought, though the animals are unique.

### 3.3. The Regional Correlation between Biodiversity and Linguistic Diversity of China

The Spearman correlation coefficient was adopted to calculate the correlation coefficients of the total numbers of plant and animal species, the numbers of plant species, and the numbers of animal species with the numbers of minority languages, and the 7 diversity indicators of Chinese dialects in the 33 PARs. The correlation between two variations is significant when the two-sided test confidence (Sig.) is less than 0.01 and the correlation coefficient (*ρ*) is higher than 0.5.

When Sig. is less than 0.01, the correlation coefficients of the total numbers of plant and animal species or the numbers of plant species with the numbers of minority language kinds, the total numbers of the 205 phonetics indicators, the total numbers of the 203 lexicon indicators, the total numbers of the 102 grammar indicators, and the numbers of the dialectical expressions for “*father*” are higher than 0.5; the correlation coefficients of the 3 indicators of biodiversity with the numbers of the regional combinations of the division of the *Rù* tone and the numbers of the regional combinations of the 8 surveyed kinship terms using reduplication are less than 0.5; and the correlation coefficients of the numbers of animal species with the numbers of minority language kinds, the total numbers of variants of the 205 phonetics indicators, and the numbers of the dialectical expressions for “*father*” are higher than 0.5. Moreover, the correlation coefficients of the 3 indicators of biodiversity with the number of the kinds of Chinese slices are less than 0.5 (Table 3).

The results demonstrated a significant positive regional correlation between biodiversity and Chinese dialect diversity, phonetic, lexical, and grammatical diversity of Chinese dialects in China. Additionally, the PRAs with high biodiversity have high diversity of Chinese dialects. Compared with the regional correlation between animal diversity and language diversity, the positive regional correlation between plant diversity and language diversity is more significant. Among the three single representative indicators of phonetic, lexical, and grammatical diversity of Chinese dialects, the number of the dialectical expressions for “*father*” presents a significant regional positive correlation with the three indicators of biodiversity. There was no regional positive correlation between the other two representative indicators of Chinese dialect diversity and the three indicators of biodiversity.

## 4. Discussions

Linguistic diversity is formed and maintained by the variations, selections, and reproductions of languages, creatures, and their interaction [11]. The dispersion of the population is conducive to the formation of language diversity. If the early ethnic groups engaged in gathering wild fruits and hunting wild animals split into two ethnic groups, language changes may lead to difficulties in language communications between the two groups and the formation of two new languages in 2–3 centuries or 8–10 generations due to rapid language changes within both groups [37]. There is an intrinsically interdependent relationship between language diversity and biodiversity [38]. The areas with high biodiversity are characterized by large terrain fluctuation, many rivers, hot and rainy climates, and high vegetation coverage. Natural barriers, such as mountains, rivers, and dense forests, hinder language communications of different ethnic groups, resulting in the differentiation of languages and the formation of linguistic diversity.

In the areas with high biodiversity, ancient ethnic groups possess diverse and plentiful food, with a large population of long-life spans, diverse production, and lifestyles. People within ethnic groups need more phonetic, lexical, and grammatical language elements to describe various plants and animals and complex natural phenomena, express their feelings, and exchange information when engaging in gathering wild fruits, hunting wild animals, adapting to the complex natural environments, avoiding the damage of wild animals, and preventing natural disasters, contributing to the promotion of the change in speech and the formation of linguistic diversity [39].

The loss of linguistic diversity was mainly caused by industrialization, urbanization, migration of population, natural disasters, and famine. At present, 83 languages are used by 80 percent of the population of the world; there are 2500 endangered languages, most of which do not have written words. One kind of language vanishes every two weeks on average. Moreover, 95 percent of the world’s existing languages are expected to go extinct, and 5 percent of languages will survive in the 21st century [9]. The languages spoken by small populations have a greater chance of extinction in biodiversity hotspots and wilderness areas with high biodiversity [13].

With the industrialization and urbanization, the regional economic and cultural exchanges in different regions are becoming increasingly frequent, and the popularizing rate of mandarin is continually increasing for effective communication in different regions. Thus, convergence changes in various Chinese dialects have occurred. Some dominant Chinese dialects have maintained competitive relationships with mandarin for a long time, in the process of declining, and some secondary Chinese dialects have been evolving into dominant ones or mandarin [40]. The phonetic, lexical, and grammatical diversity of Chinese dialects have also changed.

The chances and frequency of people using Chinese dialects and their distinctive words have decreased as a result of the popularization of mandarin and the improvement of education. The decrease has led to the gradual disappearance of some Chinese dialectal words, as well as the gradual evolution of some Chinese dialectal words into those of Chinese mandarin. For example, the Chinese mandarin word *taìyáng* (the Sun) has been expressed by 21 lexical items of Chinese dialects in Shandong [41]. Nowadays, only middle-aged and old people in some rural areas use local lexical items of Chinese dialects to indicate the Sun, while students, young people, and urban residents mainly adopt Chinese mandarin “*tài yáng*”. The population inheriting lexical items of Chinese dialects is becoming fewer than before.

Chinese dialects can arouse people’s sense of identity and belonging. People away-from-home will feel friendliness with each other when they communicate with the same dialects. The unification of Chinese dialects, the convergence of Chinese dialectal words, and the loss of their diversity have reduced cultural diversity and weakened people’s sense of belonging and identity to their hometown. Effective measures should be taken to protect the natural and social environment in which the diversity of Chinese dialects have formed, such as adopting differentiated urban development strategies in different regions, appropriately regulating the urbanization process in areas with high biodiversity, protecting local World Heritage sites, numerous small communities, and the healthy ecosystems on which the production and living patterns of the residents depend, sustainably using biological resources and protecting biodiversity. Humans should vigorously promote the national project of protecting language resources, record and preserve the existing Chinese dialects comprehensively and scientifically, and apply Chinese dialects in literary creation and artistic performances, so as to enhance the vitality of Chinese dialects.

The numbers of Chinese dialect kinds; the data of phonetic, lexical, and grammatical diversity of Chinese dialects; the animal and plant species; the plant species; and the animal species in the 33 PARs of China have been divided into five grades based on the natural differences inherent in each data series by using the natural discontinuous point method of ArcGIS10.2 software. The division can maximize the differences of data in different grades to visually exhibit their regional differences. This paper verifies the regional correlation between the phonetic, lexical, and grammatical diversity of Chinese dialects at the micro linguistic level and animal and plant diversity, plant diversity, and animal diversity, deepening the research on the regional correlation between linguistic diversity and biodiversity.

There is no regional correlation between the number of Chinese dialect kinds and the biodiversity indicators, such as the total numbers of animal and plant species, plant species, and animal species, owing to industrialization, urbanization, and population migration. Southwest China possesses high biodiversity and diversity of minority languages. The phonetics, lexicon, and grammar of Chinese dialects are relatively different there. Due to the impact of population migration to cities and interregional migration caused by urbanization, the convergence of Chinese dialects has changed rapidly and significantly, and there are only a few varieties of Chinese dialects.

The phonetic, lexical, and grammatical diversity of Chinese dialects in south-western PARs is relatively low, while the diversity of minority languages and their phonetic, lexical, and grammatical diversity are relatively high. Thus, the linguistic diversity there is high. Due to the lack of data, this paper failed to verify the regional correlation between the diversity of minority languages, and their phonetic, lexical, and grammatical diversity and biodiversity in China. If the diversity of minority languages and Chinese dialects is comprehensively considered, the regional correlation between linguistic diversity and biodiversity will be more significant.

## 5. Conclusions

There are regional differences in the diversity of Chinese dialects; the phonetic, lexical, and grammatical diversity of Chinese dialects; and the diversity of animal and plant species in China. The phonetic, lexical, and grammatical diversity of Chinese dialects has significant regional positive correlations with the diversity of animals and plants in China. The positive regional correlation between linguistic diversity and plant diversity is stronger than that between linguistic diversity and animal diversity. Moreover, the diversity of Chinese dialects is declining with the rapid industrialization and urbanization in China. Thus, countermeasures should be taken to protect the diversity of Chinese dialects.

The biodiversity and linguistic diversity are influenced by the differences in area and population of the 33 PARs. The study neglected the impact of the size of the PARs on regional differences in plant and animal species and the impact of the population of the PARs on regional differences in linguistic diversity. The data on phonetic, lexical, and grammatical diversity of Chinese dialects were collected from the survey results of 2002–2006. Hence, the data of some indicators may have changed slightly, affecting the accuracy of the results. However, the conclusions cannot be affected by these changes. In addition, this study did not include the Tibetan Autonomous Region due to the lack of survey data on Chinese dialects in that PAR.

## Figures and Tables

**Table 1:** Statistical indicators of biodiversity and Chinese dialects.

Provinces (Including Autonomous Regions, Municipalities, and Special Administrative Regions)	Totalnumber of Animal And Plant Species	Total Number of Plant Species	Total Number of Animal Species	Number of Dialect Slices	Total Number of Variants of the 160 Phonologies, Pronunciations of the 32 Special Characters, and the 13 Important Phonetic Values of Chinese Dialects *	Total Number of the Dialectical Expressions for the 203 Chinese Words	Total Number of the Dialectical Expressions for the 102 Chinese Grammatical Vocabularies, Morphology, and Syntax	Number of the Regional Combinations of the Division of the *Rù* Tone	Number of the Dialectical Expressions for the Chinese Word “Father”	Number of the Regional Combinations of the 8 Surveyed Kinship Terms Using Reduplication
Heilongjiang Province	6153	2882	3271	3	242	143	146	2	3	9
Jilin Province	5486	2837	2649	2	226	151	144	2	2	6
Liaoning Province	6042	2831	3211	5	263	166	158	2	3	7
Inner Mongolia Autonomous Region	6387	3363	3024	8	437	221	227	7	5	8
Xinjiang Uyghur Autonomous Region	8688	4617	4071	4	353	176	162	6	5	9
Hebei Province	6409	3183	3226	7	593	300	302	7	6	20
Beijing City	3530	715	2815	3	228	137	141	2	2	3
Tianjin City	1236	364	872	1	215	94	94	1	1	1
Shanxi Province	4994	2711	2283	9	710	272	269	12	6	14
Shaanxi Province	9140	5054	4086	8	647	323	320	8	6	11
Ningxia Hui Autonomous Region	3233	1511	1722	5	352	188	177	3	5	5
Gansu Province	8975	5358	3617	5	495	268	261	5	6	9
Qinghai Province	5223	2996	2227	2	311	148	167	4	4	5
Shandong Province	4761	2419	2342	8	578	260	287	9	9	13
Henan Province	5573	3138	2435	6	487	258	249	4	7	13
Jiangsu Province	5321	3006	2315	5	1069	408	502	7	9	22
Shanghai City	1968	579	1389	1	382	153	216	1	3	8
Anhui Province	5506	3701	1805	14	1289	460	554	10	18	22
Hubei Province	9034	5542	3492	6	889	373	397	6	17	15
Chongqing City	3587	2282	1305	3	257	186	184	1	4	7
Sichuan Province	22,724	13,494	9230	3	394	289	237	2	13	11
Zhejiang Province	10,524	5073	5451	10	1705	634	949	4	22	28
Fujian Province	10,947	5473	5474	6	1745	682	824	9	21	18
Jiangxi Province	7725	4843	2882	15	1707	598	731	9	16	24
Hu’nan Province	9201	5588	3613	15	1958	694	809	11	19	39
Guizhou Province	12,200	8204	3996	5	342	219	214	4	7	9
Yunnan Province	31,572	19,957	11,615	4	459	240	247	2	6	14
Guangdong Province	12,110	7387	4723	11	1537	739	856	8	19	11
Guangxi Zhuang Autonomous Region	14,937	9557	5380	8	1466	718	805	11	22	14
Henan Province	11,350	5186	6164	5	676	275	226	8	8	1
Taiwan Province	17,539	6992	10,547	2	564	227	269	5	4	2
Hong Kong Special Administrative Region	3847	1622	2225	1	303	139	142	2	2	2
Macao Special Administrative Region	1156	601	555	1	203	95	98	1	1	1

* Note: The variants of the 160 phonologies of Chinese dialects can be created by a variety of transformation methods, such as preservation, change, division, and combination.

**Table 2:** Chinese dialect districts and their corresponding dialect slices, population.

Chinese Dialect District	Sub-Dialect District	Dialect Slices	Population (×10^4^ Capita)
Mandarin district	Northeastern Mandarin sub-district	3	9802
	Beijing Mandarin sub-district	2	2676
	Ji-Lu Mandarin sub-district	3	8942
	Jiao-Liao Mandarin sub-district	3	3495
	Zhongyuan Mandarin sub-district	13	18,648
	Lan-Yin Mandarin sub-district	4	1690
	Jianghuai Mandarin sub-district	3	8605
	Southwestern Mandarin sub-district	6	26,000
Jin Dialect district		8	6305
Wu Dialect district		6	7379
Min Dialect district		8	7500
Hakka Dialect district		8	4220
Cantonese Dialect district		7	5882
Hunan Dialect district		5	3637
Gan Dialect district		9	4800
Hui Dialect district		5	330
Pinghua and Tuhua Dialect district		4	778

**Table 3:** Spearman correlation coefficients of biodiversity with the diversity of minority languages and the diversity of Chinese dialects in China.

Indicators of Biodiversity	Spearman Correlation Coefficient and the Two-Sided Test Confidence (Sig.)	Number of the Kinds of Chinese Slices	Total Number of Variants of the 160 Phonologies, Pronunciations of the 32 Special Characters, and the 13 Important Phonetic Values of Chinese Dialects	Total Number of the Dialectical Expressions for the 203 Chinese Words	Total Number of the Dialectical Expressions for the 102 Chinese Grammatical Vocabularies, Morphology, and Syntax	Number of the Regional Combinations of the Division of the *Rù* Tone	Number of the Dialectical Expressions for the Chinese Word “*Father*”	Number of the Regional Combinations of the 8 Surveyed Kinship Terms Using Reduplication
Total number of plant and animal species	Spearman correlation coefficient	0.370 *	0.542 **	0.623 **	0.558 **	0.402 *	0.628 **	0.397 *
	Two-sided test confidence (Sig.)	0.030	0.001	0.000	0.001	0.020	0.000	0.022
Number of plant species	Spearman correlation coefficient	0.434 *	0.600 **	0.688 **	0.624 **	0.463 **	0.706 **	0.477 **
	Two-sided test confidence (Sig.)	0.012	0.000	0.000	0.000	0.007	0.000	0.005
Number of animal species	Spearman correlation coefficient	0.283	0.458 **	0.516 **	0.457 **	0.328	0.515 **	0.291
	Two-sided test confidence (Sig.)	0.111	0.007	0.002	0.007	0.062	0.002	0.100

** Two-sided test confidence < 0.01 level, * Two-sided test confidence < 0.05 level.

## Data Availability

Not applicable.

## Appendix A

**Table A1:** Phonologies, pronunciations of special characters, and phonetic values of Chinese dialects surveyed by the *Linguistic Atlas of Chinese Dialects · Phonetics*.

Types of Surveyed Items	Surveyed Items
Initial, 77	Development of *Qièyùn* voiced obstruent initials, voiced obstruent initials, evelopment of *qièyùn* voiced fricative initials, voiced fricative initials, nasal and lateral initials, [ɓ ɗ ɠ] initials, [tʂ tʂ^h^ ʂ] initials, [ɬ] initial, comparison of the initials in *băi*, *pāi*, & *bái*, the initials in *pái*, *bèi*, *bìng*, and *bái*, the initials in *băo* and *bàn*, the initials in *mĭ*, the initials in *feī*, *fēng*, and *fú*, the initial in *wèn*, comparison of the initials in *fŭ* & *hŭ*, the initials in *duì* and *dēng*, the initials in *tàn*, the initials in *tong*, the distinction between the *qièyùn* dental nasal and lateral initials, comparison of the initials in *năo* & *lăo* and *nán* & *lán*, comparison of the initials in *ní* & *lí* and *nián* & *lián*, the initials in *lí*, the initials in *zăo*, the initials in *zuò*, the initials in *xiào*, the initials in *sān*, the initials in *cí* and *xiè*, the distinction between the *qièyùn* dental sibilant and velar initials before high front vowels, comparison of the initials in *jiŭ* (wine) and *jiŭ* (nine), comparison of the initials in *xuĕ* and *xuè*, comparison of the initials in *jiāng* (starch), *zhāng* and *jiāng* (ginger), comparison of the initials in *quán* (complete), *chuán* and *quán* (authority), the initials in *zhū, chōu*, and *chóng*, the initials in *shŏu*, the initials in *rè*, the initials in *ruăn*, the initials in *zhuān*, the initials in *shū*, comparison of the initials in *zhāng* (sheet), *zhuāng* and *zhāng* (seal), comparison of the initials in *shān* & *shàn*, comparison of the initials in *chāo* & *căo*, comparison of the initials in *shī* & *sī*, the initials in *jiē*, the initials in *kè*, the initials in *kāi*, the initials in *qiáo*, the initials in *áo*, the initials in *yín*, the initials in *xì*, the initials in *hòu*, the initials in *xiàn*, comparison of the initials in *huáng* & *wáng*, the initials in *ēn*, the initials in *wēn*, the initials in *yŭ*, the initials in *yuán*, the initials in *yán*, the initials in *yòng*, the initials in *pŭ*, the initials in *niăo*, the initials in *tong*, the initials in *tà*, the initials in *nòng*, the initials in *què*, the initials in *song*, the initials in *shì*, the initials in *chăn*, the initials in *shŭ*, the initials in *gū*, the initials in *gōu*, the initials in *jīn*, the initials in *jìn*, the initials in *qù*, the initials in *xī*, the initials in *guì*, the initials in *xióng*, the initials in *qiān*.
Final, 84	Development of nasal codas in finals, the codas [m n η], nasalized finals, development of final with oral stop codas, the codas [p t n l ʔ], development of *qièyùn* codas * -m and * -p, the codas in *sān* and *chā*, the codas in *xīn* and *sh**í*, the codas in *shān* and *shā*, the codas in *xīn* and *qī*, the codas in *xiăng* and *xuē*, the codas in *shuāng* and *xué*, the codas in *shēng* and *sè*, the codas in *chéng* and *chĭ*, the codas in *zhōng* and *zhú*, the final in *dà*, comparison of the finals in *gē* & *guò*, comparison of the finals in *guò* & *gŭ*, comparison of the finals in *gē* & *gāo*, the final in *chē*, comparison of the finals in *xiě* & *xié*, comparison of the finals in *zhū* & *zhŭ*, comparison of the finals in *jù* & *qū*, the finals in *shū*, comparison of the finals in *lù* & *loú*, comparison of the finals in *chū* & *choū*, the final in *kāi*, comparison of the finals in *dài* (bag) & *dài* (belt), the final in *ăi*, comparison of the finals in *pái* & *pài*, comparison of the finals in *dài* & *pái*, the final in *tì*, the final in *mèi*, the final in *léi*, comparison of the finals in *jī* & *jì*, comparison of the finals in *zhĭ* & *shī*, the finals in *pí, dì, xì* and *yī*, the finals in *fēi*, the final in *shuĭ*, the finals in *guĭ*, the finals in *hăo*, comparison of the finals in *băo* (treasure) & *băo* (satiated), comparison of the finals in *xiào* & *diào*, the final in *dòu*, comparison of the finals in *lóu* & *liú*, comparison of the finals in *fú* & *fŭ*, comparison of the finals in *nán* & *lán*, comparison of the finals in *jiē* & *tiē*, comparison of the finals in *sān* & *shān*, comparison of the finals in *tán* & *tang*, the final in *lín*, comparison of the finals in *xīn* (heart) & *xīn* (new), comparison of the finals in *shēn* & *sheng*, comparison of the finals in *xīn* & *xīng*, comparison of the finals in *miàn* (face, surface) & *miàn* (wheat flour), the final in *bàn*, the final in *suān*, comparison of the finals in *hàn* & *guan*, the final in *gēn*, the final in *cùn*, the final in *sŭn*, comparison of the finals in *gŭn* & *gong*, comparison of the finals in *yún* & *yòng*, the final in *tang*, the final in *yào*, comparison of the finals in *kāng* & *jiăng*, comparison of the finals in *táng* & *lěng*, comparison of the finals in *shuāng* & *chóng*, the final in *dēng*, the final in *sè*, comparison of the finals in *bīng* (ice) & *bīng* (soldier), the final in *zhēng*, the final in *xīng*, comparison of the finals in *dēng* & *dōng* and *yĭng* & *yòng*, the final in *hóng*, the final in *fēng*, the final in *long*, the final in *zú*, comparison of the finals in *zhōng* & *zhŏng*, comparison of the finals in *zhú* (bamboo) & *zhú* (candle), the final in *hái*, the final in *dă*, the final in *gěng*, finals with a high front rounded vowel as medial or main vowel, medials, apical vowels.
Tone, 38	Tonal distribution of contrasting codas, number of tone categories, division of the *Píng* tone, division of the *Shăng* tone, division of the *Qù* tone, division of the *Rù* tone, tonal distribution of *qièyùn* voiceless unaspirated and aspirated initials, tonal distribution of *qièyùn* voiced and sonorant initials, merges of *píng* tone sonorants, merges of *shăng* tone voiced obstruents, merges of *shăng* tone sonorants, merges of *qù* tone sonorants, development of the *rù* tone, merges of *rù* tone voiceless obstruents, merges of *rù* tone voiced obstruents, merges of *rù* tone sonorants, tone of pitch-contour of *dōng*, tone of pitch-contour of *tong*, tone of pitch-contour of *zhŏng*, tone of pitch-contour of *zuò*, tone of pitch-contour of *dòng*, tone of pitch-contour of *shù*, tone of pitch-contour of *bĕi*, tone of pitch-contour of *băi*, tone of pitch-contour of *dú*, comparison of the tone in *tóng* with *dŏng* & *dòng*, comparison of tones in *zuò* & *dà*, comparison of tones in *bĕi* & *băi*, the tone of *ting*, the tone of *aó*, the tone of *maō*, the tone of *long*, the tone of *nóng*, the tone of *Bing*, the tone of *pài*, the tone of *caō*, the tone of *bí*, the tone of *lā*, nasal and lateral syllabics.
Combination of initial and final and other specials, 4	The initial and final in *chá*, the initial and final in *wú*, the initial and final in *ér*, nasal and lateral syllabics.

**Table A2:** Vocabularies of Chinese dialects surveyed by the *Linguistic Atlas of Chinese Dialects* · *Lexicon*.

Types	Surveyed Chinese Vocabularies
Nouns, 95	Sun, Moon, thunder, rainbow, hail (noun), today, tomorrow, last year, rice (the plant), wheat straw, corn (the plant), meal/powder, sweet potato, potato, peanut, fava bean, radish, Chili pepper, eggplant, tomato, sunflower (the plant), boar (used for breeding), sow (that has farrowed), pigsty, dog, egg (of a chicken), monkey, tiger, rat/mouse, snake, bird, nest (of a bird), person, child, guest, paternal grandfather (direct address), paternal grandmother (direct address), maternal grandfather (direct address), maternal grandmother (direct address), father (direct address), mother (direct address), aunt (mother’s brother’s wife, direct address), husband (direct address), wife (direct address), sun (direct address), daughter in law (direct address), daughter (direct address), sun in law (direct address), head (of a human), face, eye(s), nose, mouth (of a human), tongue, neck, left hand, right hand, fist, foot, belly (abdomen), buttocks, excrement, penis, terms for “penis” derived from animal names, semen, breasts (of a woman), clothes, sleeve, steamed buns (unstuffed), steamed buns (stuffed), cooked dishes, euphemisms for pig’s tongue, euphemisms for pig’s liver, vinegar, village, alley, house (one ~), room (one ~), window, heated brick bed, grave (a ~), older words for cement, pot, wok (for cooking), firewood, kitchen knife, chopsticks, table, bottle, lid/cap, comb, soap (for washing clothes), umbrella, thing, thing/matter/event, vulva.
Adjectives, 29	Many, small, thick, wide (diameter) (the rope is ~), thin/narrow (diameter) (the rope is ~), tall (stature), short (stature), thick (depth) (the board is ~), thin (depth) (the board is ~), wide (the road is ~), narrow (the road is ~), curved, crooked (the road is ~), dense (the crops are planted ~ly), sparse (the crops are planted ~ly), bright (of light), black (the color), hot (of the weather), cold (of the weather), bland/insipid (this food is ~), painful (took a ~ fall), dry (the clothes are ~), late (come late), fat (of pigs), fat (of people), thin (of people and pigs), beautiful, pretty (she is ~), correct (the account was calculated ~ly), incorrect (the account was calculated ~ly), hungry, thirsty.
Verbs, 53	Castrate (a boar), butcher (a pig), rain (verb), lay (an egg), fuck, wear (shoes), tie (shoelaces), take off (shoes), eat (a meal), drink (wine), pick up (food with chopsticks), pour (wine), boil (eggs), deep-fry (dough sticks), watch (television), smell (with the nose), say, call out (after him), cry, scold (someone), bite (the dog bites someone), hit, hold (a child) in one’s arms, uproot/pull out (a radish), grab (a theif), carry (a sedan chair), carry (with a shoulder pole), stand (up), squat (down), jump, step on (cow dung), walk, run, escape, wipe (one’s hands dry), chop down (a tree), bury, hide, put (the bowl on the table), fall (down), pick up, find, select, owe, give, want (I want this one), think, know, be afraid, play/have fun, take a bath, sleep, marry (a woman).
Numerals, 3	One, two, twenty
Adverbs, 2	Sharp (the knife is sharp), fast (it is ~er to take the bus than to walk)
Quantifiers, 10	Measure word for people, measure word for pigs, measure word for dogs, measure word for trees, measure word for autos, measure word for matters, business, measure word for written characters, measure word for meals, measure word for basic monetary unit, dollars, measure word for ten cents.
Special vocabularies, 11	Semantic range of *wénzi* (mosquito), comparison of terms for *wàisūn* (daughter’s son) & *wàisheng* (sister’s son), semantic range of *sh**ŏu* (hand) and *ji**ăo* (foot), comparison of terms for *lā* (defecate) & *sā* (urinate), comparison of terms for *hē* (drink) & *chī* (eat), semantic range of *fáng* (house), semantic range of *wū* (room), semantic range of *cháng* (long), semantic range of *xì* (thin), comparison of terms for *pàng & feí* (terms for fat), idioms referring to “the Moon” as a person.

**Table A3:** Chinese dialects expressions of grammatical words, morphology, and syntax surveyed by the *Linguistic Atlas of Chinese Dialects* · *Grammar*.

Map Types		Surveyed Items
Maps of Structures, 51	Maps of standard Chinese forms, 29	The suffix in *zhuōzi* (table), perfective aspect with substantive object (*wŏ chī le yī wăn fàn*, I ate a bowl of rice), the passive construction (*wăn bèi tā dă pòle*, bowl is broken by him), the negative comparative (*wŏ meíyou tā dà*, I am not elder than him), the noun suffix *ér* (including rhotacized forms), the suffix in *jiàohuāzi* (beggar), the suffix in *niăoer* (bird), suffixes derived from kinship terms, perfective aspect *le*_1_ and sentence particle *le*_2_ (*tā laíle sān tiān le*, he has been here for three days), perfective aspect *le* without object (*tā laíle*, he is coming), progressive aspect (he is having dinner), continuous aspect, the existing progressive *zhe*, potential complement *dé*, potential complement *bùdé*, the result complement *qĭ lái* in *nĭ zhàn* ~ (stand up), word order in *bùzhīdào* (don’t know),word order *qù* in *wŏ măi cài ~* (I’m am going food shopping), the order of object and affirmative potential complement (*dă de guò tā*, beat him), the order of object and negative potential complement (*dă bū guò tā*, can’t beat him),the order of object and directional complement (*xià kāi yŭ le*, it’s raining),the order of object and quantity complement (*jiào tā yī shēng*, call him), word order in *nĭ xiān qù* (you go first), word order in *zài chī yīwăn* (have another bowl [of rice]), the disposal construction (*tā bă wăn dă pòle*,he broken the bowl), double-object imperative constructions (*gěi wŏ yī zhī bĭ*, give me a pen/pencil), the comparative (*wŏ bĭ tā dà*, I am elder than him), *qù bū qù ?* affirmative-negative question (*míng tiān nĭ* ~, will you go tomorrow ?), *qù méi qù ?* affirmative-negative question for the perfective aspect (*zuó tiān nĭ* ~, did you go yesterday ?)
	Maps of Dialect Forms, 22	Measure words as demonstratives, the noun suffix *jiăn*, *xiān* as postposition marking preceding action in *nĭ qù ~* (you go first), degree adverb *hĕn* (very) in post adjective position, the familiarizing prefix *ā* used in names, the familiarizing prefix *ā* used in kinship terms, the kinship prefix *lăo*, the prefix *gē*, the noun suffix *tóu*, the suffix *tóu* used to denote monetary amounts, the noun suffixes *zăi* and *zăi*, reduplicated single-syllable nouns, reduplicated single-syllable verbs *wènwen* (ask), reduplicated single-syllable verbs with complements *nĭkànkanqīngchŭ* (look at this clearly), reduplicated single-syllable adverbs of extent (*jīn tiān hěn hěn rè*, it is very hot today), the result complement diao in *jī sĭ ~ le* (the chicken died), *Yŏu* as perfective verbal prefix in *wŏ ~ qù* (I went), *zhe* as postposition marking complement of one action as prerequisite to another in *xiē yīhuìr ~ zài shuō* (rest a bit and then decide), *tiān* as postposition marking additional action in *chī yīwăn* ~ (have another bowl [of rice]), *tiān* as postposition marking remaining quantity in *hái yŏu shílĭ lù* ~ (there are still 10 miles to go), *guò* as postposition marking repetition in *huàn yījiàn ~* (change into another item [of clothing]), *kàn* as postposition marking a try in *wènwen* ~ (try asking)
Maps of Grammatical Words, 39		First-person singular pronoun *wŏ* (I) (~ *xìng wáng*, my family name is *wáng*),Second-person singular pronoun *nĭ* (You),Third-person singular pronoun *tā* (he, she), First-person plural pronoun (inclusive of listener) *zánmen* (we), the quantifier *liă* (two people), attributive of acquaintance of *wŏ bà* (my dad), reflective pronoun *zìjĭ* (myself), demonstrative pronoun *zhè* (this), demonstrative pronoun *nà* (that), interrogative personal pronoun *nă* (which), interrogative personal pronoun *shuí* (who), interrogative pronoun *shénme* (what), interrogative quantity pronoun *duōshăo* (how many/much), interrogative adverb *zĕnme* (how), degree adverb *hĕn* (very) for adjectives, degree adverb *hĕn* (very) in post adjective position, the plain negative *bù* (not), the possessive particle *de*, prefixes with the connotation “foreign”as *yáng*, the pronoun *dàjiā* (everybody), emonstratives as sentence subject, degree adverb *hĕn* (very) in extent complement construction, degree adverb *zuì* (most) for adjectives, the adverb of immediately *jiù* (then), the adverb *yòu* (again), the adverb *yĕ* (also), the adverb *fănzhèng* (in any case), the negative perfective *méiyŏu* (did not), the comparative marker *bĭ* (*wŏ ~ tā dà*, I am elder than him), the existential negative *méiyŏu* (does not have), the negative imperative *bié* (donʼt), the preposition *cóng* (from), verb of location *zài* (to be in, at, on), locative preposition *zài* (in, at, on), locative complement *zài* (into, onto), the copula *shì* (to be), the nominal conjunction *hé* (and), he locative suffix *shàng*, the disposal marker *bă* (*~ yīfu shōu huílai*, take the clothes back), the passive marker *bèi* (*yīfu ~ tōu zŏule*, clothes are stolen)
Maps of Summary, 12		Plural forms of personal pronouns, the pronoun *dàjiā* (everybody), negative word types, negative morpheme types, diminunatives, animal gender constructions, demonstrative pronoun types, the copula *shì* (to be) used as locative, kinship terms using reduplication, comparison of grammatical markers *gěi*, *bă* & *bèi*, comparison of particles used for imminent aspect and completed aspect, affirmative potential complement, negative potential complement
